# Hypoxia Treatment of *Callosobruchus maculatus* Females and Its Effects on Reproductive Output and Development of Progeny Following Exposure

**DOI:** 10.3390/insects7020026

**Published:** 2016-06-17

**Authors:** Yan Yan, Scott B. Williams, Dieudonne Baributsa, Larry L. Murdock

**Affiliations:** 1 Department of Entomology, China Agricultural University, Beijing 100193, China; yanyancau@hotmail.com; 2 Department of Entomology, Purdue University, West Lafayette, IN 47907, USA; dbaribut@purdue.edu (D.B.); murdockl@purdue.edu (L.L.M.)

**Keywords:** *Callosobruchus maculatus*, hypoxia, reproduction, egg development, hermetic storage

## Abstract

Modified atmospheres present a residue-free alternative to fumigants for controlling postharvest pests of grain during storage. How sub-lethal applications of this method affects the reproductive fitness of target pests, however, is still not fully understood. We examined how low levels of ambient oxygen influence the reproduction of the female cowpea bruchid (*Callosobruchus maculatus*), a pest of cowpea. We used three low-oxygen atmospheres—2%, 5% and 10% (*v*/*v*) oxygen—and observed their effects on: (1) the number of eggs laid by bruchids compared to insects held in normoxic (~20% oxygen) conditions; (2) the total number of eggs laid; and (3) the number of progeny that reached maturity. Low oxygen did not significantly affect the number of eggs laid during 48 or 72 h of exposure, but 2% and 5% oxygen did negatively affected total egg production. Increasing the exposure time from 48 to 72 h further depressed lifetime reproductive output. Maternal and egg exposure to hypoxia reduced the number of progeny that reached adulthood. Lower adult emergence was observed from eggs laid under low oxygen and longer exposure times. These data demonstrate that hermetic conditions depress the egg-laying behavior of cowpea bruchids and the successful development of their progeny.

## 1. Introduction

The cowpea bruchid, *Callosobruchus maculatus* (F.) (Coleoptera: Chrysomelidae), is a postharvest pest that feeds primarily on legume seeds as larvae [1]. In Africa, *C. maculatus* is a serious pest on cowpea, a major staple and cash crop [2]. Cowpea is infested by *C. maculatus* in the field [3], but its populations grow exponentially when the grain is threshed and stored after harvest. This can result in total loss after a few months of storage [4]. Infested cowpeas lose nutritional value and germination viability and can be rendered unsuitable for human consumption and planting [5].

Fumigants like methyl bromide and phosphine have been used to keep cowpea free of bruchids and other arthropod pests in storage. However, the wide use of synthetic insecticides has encouraged selection for resistant traits among insect pests. This has added to the cost of pest control and increased environmental pollution [4,6,7,8,9,10]. In sub-Saharan Africa, suitable insecticides are not widely available nor are farmers trained in their use.

Modified atmospheres (MA) can provide an efficient, residue-free alternative to fumigants and other chemicals for postharvest pest control [11,12,13,14]. MA technologies, like the Purdue Improved Crop Storage (PICS) bags, have been effective in stopping losses to insects in stored grains including cowpea, maize, and groundnut [15,16,17,18]. The PICS bag consists of two inner liners of high-density polyethylene (HDPE) and an outer, woven layer of polypropylene that makes the compound bag durable when handled [16,19]. PICS bags have been disseminated widely among smallholder farmers in tens of thousands of villages across West and Central Africa for postharvest storage of cowpea and, more recently, for other dry grains [20,21,22,23].

While PICS bags and other hermetic containers suppress the development of bruchid populations by depriving them of oxygen, the details of the protective action are still not completely understood [16,24]. By blocking the supply of oxygen, essential both for energy production as well as for the supply of water required by insects that live in extremely dry environments, PICS bags suppress feeding, growth, development, and population expansion. One effect of a reduced supply of oxygen is that reproductive behavior may be impaired when female bruchids lack the necessary metabolic resources to complete egg development. An effect on reproduction has already been observed in systems with elevated carbon dioxide [25]. However, few studies have specifically observed the behavior of cowpea bruchids at oxygen levels between 2% and 10% of the total atmosphere. This range represents the typical, internal oxygen levels observed in sealed PICS bags after the respiration of the insects has depleted much of the available oxygen [23].

In the present study, we sought to shed light on the effect of hypoxic atmospheres on the reproductive behavior of *C. maculatus*. We sought to determine (1) the effect of lowered oxygen levels (2%, 5%, and 10% of total atmosphere) on the number of eggs laid by adult female cowpea bruchids; and (2) the effect of exposure on eventual adult emergence from eggs laid by female bruchids under hypoxia.

## 2. Materials and Methods

### 2.1. Insect Colonies

Colonies of *Callosobruchus maculatus* originated from insects collected in Niger, West Africa. They were maintained at the Department of Entomology, Purdue University on California black-eyed cowpea seeds variety #8046 (Wax Co., Amory, MS, USA) and stored in a Conviron^TM^ Environmental Chamber (C710, Winnipeg, MB, Canada) at 25 °C and 40% R.H.

Cowpea bruchid colonies were set up by putting ~150 adult bruchids in a 500 mL wide-mouth glass jar, filled approximately ¼ full with fresh cowpea seeds. The insects were allowed to lay eggs for two hours and then removed. Eggs were allowed to develop under the above temperature and humidity conditions and 12:12 LD cycle to maturity with the time of first adult emergence monitored.

### 2.2. Baseline Bruchid Reproductive Output

When preparing for the baseline reproductive output study, a colony was set up and monitored as described above. When adult bruchids were first observed in this jar, all adult insects were removed via sifting with a No. 10 sieve. After 24 h, we selected female bruchids that subsequently emerged. Selection was based on the length of the abdomen beyond the elytra. For female bruchids, this length is greater than 1 mm and gives the insect an ovoid shape, while for males, it is less than 1 mm and gives the insect a rounded appearance [26,27,28]. Ten female cowpea bruchids were collected from the colony jar. Each female was held in the absence of cowpea oviposition sites with two males in a 30 mL glass bottle for 12 h to ensure mating. Messina *et al.* [29] observed that male and female cowpea bruchids mate readily and with great predictability (>74% chance of copulation) within only a few minutes’ time in the same enclosure. This arrangement allowed us to be confident in the female’s mated status for our trials, even if direct observation of the event was not made. After 12 h, the female bruchid was transferred via vacuum aspirator into a second, 30 mL glass bottle containing five, fresh cowpea seeds. Each bottle was sealed with a plastic lid with holes to allow access to fresh air.

After 24 h, the females were transferred to new bottles containing five, fresh cowpea seeds in each. The eggs laid during the first 24 h were recorded and labeled as Day 1. This process was repeated every 24 h (Day 2, Day 3, *etc.*) until all insects had died. Seeds were held as separate cohorts and monitored daily for 60 days. We collected data on the length of time that passed before the first adults emerged from each cohort and the number of adults after 60 days.

### 2.3. Effect of Reduced Oxygen on Bruchid Egg Production

All adult bruchids were removed from a colony jar three days after the first adults emerged. The colony jars were held for an additional 24 h. All adults that emerged during this period were isolated in separate jars for 24 h before being used for the experiment. This step ensured that all females used in the trial were between 2 and 3 days old. In total, 36 adult females were collected from these jars for each trial.

The gravid females were placed individually into 30 mL glass vials, each containing 5 cowpea seeds. Each vial was covered with a silicone cap perforated with air holes to allow gas exchange. All vials were placed into an airtight, gas chamber. Desired oxygen levels (2%, 5%, and 10%) were obtained by flushing the chamber with nitrogen from a compressed-gas cylinder. Oxygen levels were continually monitored using a 5250i Oxysense monitor (OxySense^®^, Las Vegas, NV, USA) until the desired concentration was reached, after which it was maintained within ±1% of the chosen treatment values. 

For each trial, female bruchids were exposed to one of three oxygen environments (2%, 5%, or 10% oxygen) for either 48 or 72 h. Following that exposure, the insects were transferred to new vials, each containing 5 uninfested cowpea seeds. The vials were held in normoxic (oxygen equals approximately 20% total atmosphere) conditions for another 48 or 72 h period. We repeated the transfer of female bruchids into a vial with fresh cowpea seeds and held them under normoxic conditions one additional time. Each treatment cohort contained 12 female bruchids (24 per trial) and after each transfer, we counted the number of eggs each female laid during that period. The final counts allowed us to determine the total number of eggs laid during exposure to different oxygen levels as well as over their adult lifetime. 

We also tracked the development of eggs laid by the treated female bruchids. We did this by monitoring the number of insects that reached adulthood and the length of time required. Eggs from the same individual female and laid during the same period were isolated from other cohorts using separate containers. Final assessment of adult emergence from these eggs was performed after 60 days. For each treatment group, a concurrent control group that had not been exposed to hypoxia was also set up, with female reproductive output assessed in the same manner as described above. 

### 2.4. Statistical Analysis

For the baseline reproductive study, the number of eggs laid and percentage of progeny that develop into adults were compared statistically using Analysis of Variance (ANOVA) to determine if reproductive rates were different depending on the age of the female bruchid. Comparisons of average reproductive output and final adult emergence between treatment and control groups was performed using a Student’s *t-*test (2-sample, equal variance, *p* < 0.05). When needed, comparisons between the two, exposure-time treatments (48 and 72 h) were also compared using Student’s *t*-test.

## 3. Results

### 3.1. Baseline Reproductive Output of Female Bruchids

All female cowpea bruchids remained alive for at least four days, after which we began to observe mortality, as can be seen in Table 1. All insects were dead by the 9th day. The number of eggs laid was highest on the first day (15 ± 2 per female) after emergence. Reproductive output declined gradually thereafter.

Average development time for bruchid adults from the eggs laid on Days 1 and 2 was 47 days and gradually increased with time. Eggs laid on Day 6 needed 54 days to develop to adulthood. No adults emerged from eggs laid after seven days. The greatest number of adults emerged from eggs laid on Day 1 and declined with time. Of the eggs laid between Days 1 and 5, the cumulative adult emergence ranged between 48% and 67% after 60 days. Overall, it appears that eggs laid earlier in a female reproductive cycle produce offspring that develop more quickly and with higher rates of survival than those eggs laid later (ANOVA: *F* = 13.83, d.f. = 8,89, *p* < 0.001).

### 3.2. Effect of Reduced Ambient Oxygen on Bruchid Egg Production

**Figure 1:**
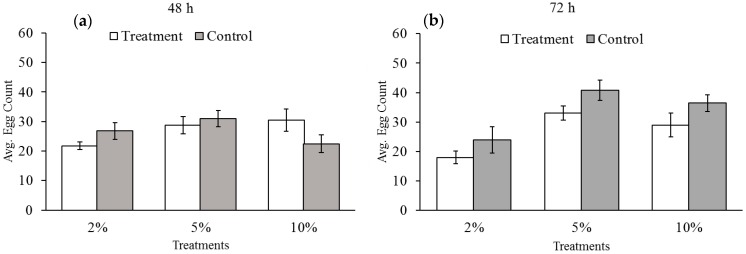
Mean number of eggs laid by female cowpea bruchids during (**a**) 48 and (**b**) 72 h of exposure to hypoxia. Asterisks represent statistically significant differences between oxygen treatment groups (white) and paired control groups (gray). Bars with no asterisk were not significantly different from the control group. Oxygen levels had no effect on the number of eggs laid relative to the control groups with the exception of the 10% treatment group exposed for 48 h.

**Figure 2:**
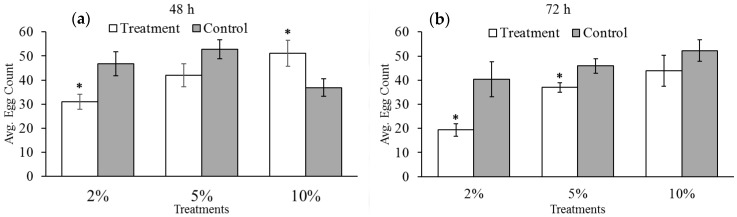
Average number of eggs laid by female cowpea bruchids over their lifetimes both during and following exposure to (**a**) 48 and (**b**) 72 h of exposure to hypoxia. Asterisks represent statistically significant differences between oxygen treatment groups (white) and paired control groups (gray). Bars with no asterisk were not significantly different from the control group. We observed negative effects of our treatment oxygen levels on the reproductive output of female bruchids relative to our control groups.

In Figure 1, we see that none of the treatments significantly reduced the number of eggs laid at the time they were exposed to reduced levels of oxygen for either 48 or 72 h (48 h: 2% *t*-test = −1.65, d.f. = 22, *p* = 0.114; 5% *t*-test = −0.54, d.f. = 22, *p* = 0.595; 10% *t*-test = 1.64, d.f. = 22, *p* = 0.115; 72 h: 2% *t*-test = −1.19, d.f. = 22, *p* = 0.246; 5% *t*-test: −1.82, d.f. = 22, *p* = 0.085; 10% *t*-test = −1.49, d.f. = 21, *p* = 0.001). However, the total number of eggs laid over their lifetimes was affected by hypoxia exposure, as seen in Figure 2. Exposure to two percent oxygen reduced total egg counts after 48 h of exposure (*t*-test = −2.69; d.f. = 18; *p* = 0.015). Increasing the length of exposure to hypoxia from 48 to 72 h further reduced the number of eggs laid (*t*-test = 2.69; d.f. = 22; *p* = 0.0103). Five percent oxygen reduced total egg count relative to the control groups, but only after 72 h of exposure (*t*-test = −2.45; d.f. = 22; *p* = 0.023) and not after 48 h (*t*-test = −1.75, d.f. = 22, *p* = 0.094).

The number of eggs laid under 10% oxygen increased relative to the control group after 48 h (*t*-test = 2.17; d.f. = 22; *p* = 0.041). This was confirmed in a follow-up trial. However, no statistical difference was observed between the 10% treatment and control groups after 72 h of exposure, suggesting the effect occurs only for shorter periods of exposure (*t*-test = −1.07; d.f. = 22; *p* = 0.297). There was also no statistical difference in the total number of eggs laid when the female bruchids were exposed to 10% oxygen for 48 h *versus* 72 h.

### 3.3. Adult-Emergence

**Figure 3:**
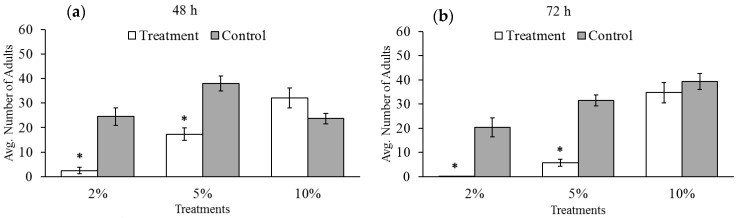
Average number of progeny emerged from bruchid eggs laid by female bruchids exposed to low oxygen for (**a**) 48 and (**b**) 72 h and under normal oxygen. Asterisks represent statistically significant differences between oxygen treatment groups (white) and paired control groups (gray). Bars with no asterisk were not significantly different from the control group. The 2% and 5% oxygen treatments significantly reduced progeny development, while 10% oxygen did not result in the same effect.

As seen in Figure 3 and Table 2, fewer adults emerged from eggs laid under 2 and 5% oxygen after both 48 and 72 h of exposure than in the controls. Increasing exposure time did not change the number of adults that emerged from eggs exposed to 2% oxygen (*t*-test = 1.77; d.f. = 22; *p* = 0.09). This is probably due to the fact that the numbers of emerged adults in either group were already low. Increasing exposure time did reduce the number of emerging adults when exposed to 5% oxygen (*t*-test = 4.14; d.f. = 22; *p* < 0.001). The longer exposure time reduced the number of larvae that survived to adulthood by an average of seven beetles. There was no difference between the number of adults that emerged under 10% oxygen and controls after either exposure time nor was there any difference between the time treatments (*t*-test = 0.51, d.f. = 22, *p* = 0.62).

## 4. Discussion

Two or three days’ exposure to low oxygen during the peak period of female bruchid reproductive activity did not reduce the number of eggs laid relative to the controls. Yet, exposure to less than 5% oxygen had negative effects on the total number of eggs laid. This, by itself, is not surprising as several studies have shown that low oxygen can impact several insect physiological systems [30,31,32,33,34]. But the effect of our oxygen treatments wass most evident after the insects return to normoxic conditions. Depending on the oxygen level and the length of exposure to subnormal oxygen, total reproductive rates over an insects’ lifespan could fall by 18%–50% compared to controls. 

This delay in the onset of reduced egg production hints at a more-complex, physiological response from the females to exposure to low-oxygen. Hypoxia inducible factor (HIF) is known to suppress protein synthesis under stressful environmental conditions [35,36,37,38]. Such biochemical changes would typically lag behind the initial stimulus, with their effects continuing well after the stimulus has been removed. Lum [39,40] and others [41] have shown that stressful environmental conditions can negatively impact a number of organ systems, including reproduction. Among the effects is the reabsorption of oocytes for resource recapture, as seen with *Plodia interpunctella* [39] and reduced oviposition in *Ephestia kuehniella* [41]. 

Cheng *et al.* [38] observed that female bruchids slow down or halt oviposition below 2% oxygen, but recover their normal rate of egg laying after 96 h in normoxic conditions. These changes mirror shifts in the insects’ transcriptional and proteolytic activities [38]. Our study is consistent with these observations. We show that while female bruchids can recover from exposure to low oxygen, there is a long-term cost in their overall reproductive output that continues even when the insects are returned to normal oxygen.

The effect of hypoxia on egg survival is also important for hermetic storage. Previously, Ofuya and Reichmuth [42] demonstrated that 100% nitrogen environments are lethal to bruchid eggs. Cheng *et al.* [38] observed that even exposure to 2% oxygen over a two day period leads to 100% egg mortality. This agrees well with our own observations. Our results indicate that the minimum oxygen level needed for an effect on reproductive success is 5% oxygen when the exposure period is three days. These same negative effects may also occur with higher oxygen levels if the exposure time is increased, but additional research is needed. 

The high egg mortality we observed is likely tied to the size and structure of insect eggs. Like many insects, bruchid eggs have a high surface area to volume ratio, which favors water loss [43]. Increased demand for oxygen under hypoxic conditions causes the egg’s spiracles to open wider, which leads to rapid water loss. Given that hypoxia can limit water availability [16], hypoxia-induced desiccation could contribute to egg death. Eggs laid by females exposed to 2%–5% oxygen saw between 57% and 100% fewer adults eventually emerge than those laid by paired control insects living in normal oxygen conditions.

For hermetic storage containers like PICS triple-bags, these data suggest that successful control of postharvest pests involves more than simply killing the insects. For cowpea bruchids, low oxygen, (5% of the atmosphere and below) may be enough to slow the rate of oviposition and embryonic development. The result would be slower growth for the insect population stored with the grain. This negative effect on reproduction would increase with the length of exposure to hypoxia. Over longer storage times, this reduced reproduction and survival would result in differences in the quality of stored grain, as slow population growth would translate to fewer seeds damaged by developing bruchid larvae. This effect works well for hermetic products like the PICS bag, which is principally used for long-term storage. In short, moderate hypoxia contributes to pest control in hermetic containers. 

## 5. Conclusions

Our study demonstrates that low oxygen (2%–5%) can suppress egg laying behavior and adult emergence of cowpea bruchids. These data support the hypothesis that hypoxia in hermetic storage can effectively control the reproductive performance of pests and so contribute to the protection of grain. However, this reproductive effect is enhanced when hypoxia is maintained for relatively long periods. Field observations in Niger show that levels of oxygen in PICS bags used to store cowpea often reach the 5–10 percent level and are maintained for weeks or months at a time [44].

## Figures and Tables

**Table 1:** Results of daily monitoring of egg laying by female *C. maculatus* under normoxic conditions. Female bruchid survival and the daily average number of eggs produced by surviving females were recorded *.

Day	Eggs Number	No. of Parent Adults Alive	First Adult Emerged (days)	Total Emerged Adults (60 days)	Percentage Emerged (%)
1	14.8 ± 1.6	10	47	7.6 ± 1.2	51.4 ± 6.2
2	10.4 ± 1.2	10	47	6.9 ± 1.0	66.4 ± 5.9
3	8.5 ± 1.2	10	48	5.5 ± 0.9	64.7 ± 3.4
4	6.0 ± 1.8	10	49	2.9 ± 0.9	48.3 ± 9.2
5	3.0 ± 1.1	7	49	1.7 ± 0.6	56.7 ± 11.5
6	2.1 ± 1.2	5	54	0.6 ± 0.3	28.6 ± 7.2
7	0.4 ± 0.3	4	NA	0 ± 0	0.0 ± 0
8	0.2 ± 0.1	3	NA	0 ± 0	0.0 ± 0
9	0.0 ± 0.0	0	NA	0 ± 0	0.0 ± 0

* Egg number, Development Time, Emerged Adults, and Percentage Emerged values are averages of cohorts laid by ten female bruchids selected for this study.

**Table 2:** Results of statistical comparisons between treatment groups and controls regarding the number of adults emerged from eggs laid by female cowpea bruchids. Hypoxia below 5% was sufficient to reduce eventual adult emergence relative to the controls.

Oxygen (%)	Exposure (h)	*t*-Value	d.f.	*p*
2	48	−5.18	13	<0.001
	72	−5.18	11	<0.001
5	48	−5.25	20	<0.001
	72	−9.73	18	<0.001
10	48	1.84	16	0.085
	72	0.70	21	0.492

## Abbreviations

The following abbreviations are used in this manuscript:

MA	Modified Atmosphere
PICS	Purdue Improved Crop Storage
HDPE	High-density polyethylene
ANOVA	Analysis of Variance
HIF	Hypoxia Inducible Factor
